# Incidence and Risks of HIV Infection, Medication Options, and Adverse Effects in Accidental Needle Stick Injuries: A Narrative Review

**DOI:** 10.7759/cureus.51521

**Published:** 2024-01-02

**Authors:** Raegan B Abadie, Elise M Brown, John R Campbell, Ivan A Alvarez, Varsha Allampalli, Shahab Ahmadzadeh, Giustino Varrassi, Sahar Shekoohi, Alan D Kaye

**Affiliations:** 1 School of Medicine, Louisiana State University Health Sciences Center, Shreveport, USA; 2 Department of Anesthesiology, Louisiana State University Health Sciences Center, Shreveport, USA; 3 Pain Medicine, Paolo Procacci Foundation, Rome, ITA

**Keywords:** hiv transmission, healthcare, postexposure prophylaxis, accidental, needlestick, hiv

## Abstract

Accidental needle sticks can lead to infections, including HIV. As scientists have learned more about HIV and its replicative physiology, identification of target sites and novel medications have been developed. HIV is spread throughout the population through contact with blood, semen, and rectal or vaginal secretions of infected individuals. Therefore, it is important in general for healthcare workers to be aware of its transmission modes and ways to minimize exposure. In this regard, even with hospitals providing education, training, and safety protocols, there is a continued infection spread with HIV, especially by accidental needle sticks. There is also a wide variety of testing that can be used for HIV utilizing different methodologies, allowing for improved measurement of infection status. Any person with HIV should be tested to clarify infection status and be educated to minimize future virus spread. The current CDC recommendations for HIV infection treatment are antiretroviral therapies, such as an HIV postexposure prophylaxis regimen, which consists of a cocktail of antiretrovirals and postexposure prophylaxis immediately for occupational exposures, such as accidental needlestick exposure from an HIV infected patient. To decrease accidental HIV stick injuries, there are safety precautions in place, that if followed, would help reduce this incidence. HIV accidental needle stick injuries still happen in the hospital workplace, but with proper education and treatment, if exposed, there is hope to minimize the effects.

## Introduction and background

An HIV infection is systemic; it initially suppresses the immune system and over time can chronically involve every organ in the body [1]. HIV is a diploid RNA virus that belongs to the retroviridae family. These viruses have an RNA genome and a reverse transcriptase, which allows these viruses to synthesize DNA via reverse transcription to be integrated within the host cell chromosomes [2]. Once the DNA is made, it can be integrated into the host cell genome and replicate further to make more viral particles. HIV establishes an infection by infecting monocytes, dendritic cells (DCs), among other CD4+ cells on mucosal surfaces [3]. HIV is a spherical virion comprising a lipid envelope containing a capsid that holds the single-stranded RNA (ssRNA) viral genome and other virion components like the nucleocapsid, integrase, and reverse transcriptase protein [4]. HIV has two glycoproteins gp41 and gp120 that are embedded in the lipid envelope of the virion and allow for entry of the virus into the host cell [2]. The HIV virus-host CD4 T-cells interaction can be achieved via the viral gp120 binding and interaction with membrane coreceptor CXCR4 (for T-cells) or CCR5 (for macrophages), resulting in conformational changes which allow the virion to fuse its gp41 to the host cell membrane, facilitating entry and the release of capsid containing the viral core proteins and ssRNA into the CD4 T-cell [2]. The HIV reverse transcriptase uses host nucleotides to make double-stranded DNA (dsDNA), and integrase allows the newly formed dsDNA to be integrated into the host chromosome. As the CD4 T-cell continues to make proteins with its own cellular DNA, it will now form more HIV RNA as well as the capsid protein, reverse transcriptase, integrase, and protease to form more virion particles to be released from the CD4 T-cell to infect other cells [3].

HIV infects individuals after coming in contact with blood, semen, and rectal or vaginal secretions of an HIV-infected individual with a mucosal surface through various modes of transmission [5].

Individuals are commonly infected with HIV through either horizontal or vertical transmission. Vertical transmission of HIV occurs from transmission from HIV-positive mother to baby in three ways: in utero, intrapartum due to interaction with secretions during birth, or postpartum via breastfeeding [6]. Typical causes of horizontal transmission involve needle usage and unprotected sexual intercourse [6]. Accidental needle stick injuries are common, estimated in the United States to be nearly 600,000 annually as almost half of the occurrences are not reported [7]. In the United States, most people who have developed HIV related to needle stick injuries have worked in the medical field, which could include nurses, laboratory workers, non-surgical physicians, or even medical students. Healthcare workers may interact with HIV patients throughout their careers, increasing the risk of being exposed to HIV. The estimated per-act risk of HIV transmission via needle injury has been noted to be 0.3%, meaning three out of every 1000 healthcare individuals who had occupational exposure to HIV-positive blood would develop HIV [8]**.** According to the CDC as of December 31, 2013, 58 confirmed occupational transmissions of HIV and 150 possible transmissions had been reported in the United States with the last confirmed case of HIV from a needle stick injury occurring in 1999[9]. 

## Review

HIV medications and side effects

For the treatment of HIV, there are currently over 30 antiretroviral drugs that are classified based on the mechanism of action [10]. The classifications currently include nucleoside/nucleotide reverse transcriptase inhibitors (NRTIs), non-nucleoside reverse transcriptase inhibitors (NNRTIs), protease inhibitors (PI), integrase inhibitors (INSTIs), fusion inhibitors (FIs), and two entry inhibitors (EIs), one blocking chemokine receptor CCR5, the other inhibiting viral interaction with CD4 [10]. Each inhibitor works at a different point in the transcription process of the HIV virion to prevent further replication of the virus. The current guidelines recommend the use of two NRTIs in combination with an integrase inhibitor or NNRTI [10]. Most commonly, the combination of drugs begins with abacavir/lamivudine, tenofovir alafenamide/emtricitabine, or tenofovir disoproxil fumarate/emtricitabine [10]. The INSTI implemented is either bictegravir, dolutegravir, or raltegravir or an NNRTI added, such as efavirenz [10]. Due to issues with medication compliance, combinations of anti-retroviral therapies have been made to improve medication adherence in patients with HIV by providing patients with single tablets and one combination of pills (Table 1 and Table 2) [11].

**Table 1:** Single-tablet FDA-approved HIV medications. Sources: [12-14] NRTI: nucleotide reverse transcriptase inhibitor; NNRTI: non-nucleoside reverse transcriptase inhibitor; PI: protease inhibitor; PK: pharmacokinetic; PKE: pharmacokinetic enhancer; EI: entry inhibitor; INSTI: integrase inhibitor; SubQ: subcutaneous; IM: intramuscular

Class	Medication name	Brand	Dosage	Side effects	Drug-drug Interactions
NRTI	Abacavir	Ziagen	300 milligrams (mg) twice a day (BID)	Hypersensitivity reaction, nausea vomiting diarrhea	*Contraindicated in patients who test positive for HLA-B5701
NRTI	Emtricitabine	Emtriva	200 mg every day (QD)	Skin discoloration and hyperpigmentation	
NRTI	Lamivudine	Epivir	150 mg BID or 300 mg QD	Headache, nausea, diarrhea, pancreatitis	
NRTI	Tenofovir disproxil fumarate	Viread	300 QD	Osteoporosis, nephrotoxicity, gastrointestinal discomfort, metabolic disorder	
NRTI	Zidovudine	Retrovir	300mg BID	1. Bone marrow suppression, severe anemia, or neutropenia; 2. Gastrointestinal discomfort: nausea, vomiting, diarrhea, and others; 3. Elevated creatinine phosphokinase and alanine transaminase, lactic acidosis, and/or hepatic steatosis	
NNRTI	Doravirine	Pifeltro	100 mg BID	Nausea, dizziness	
NNRTI	Efavirenz	Sustiva	400 mg QD	1. Central nervous system toxicity like dizziness, headache, insomnia 2. rash 3. hepatotoxic	
NNRTI	Etravirine	Intelence	200 mg BID		
NNRTI	Nevirapine	Viramune (XR)	200 mg BID	1. Rash 2. hepatoxic	
NNRTI	Rilpivirine	Edurant	25 mg QD	1. Depression 2. headache 3. insomnia	
PI	Atazanavir	Reyataz	300 mg once daily plus ritonavir 100mg once daily or atazanavir 300 mg once daily plus cobicistat 150 mg once daily.	Renal stone development and kidney injury Jaundice	
PI	Darunavir	Prezista	800 mg once daily plus ritonavir 100mg once daily or 800 mg once daily plus cobicistat 150 mg once daily.	Drug-induced liver injury	
PI/PKE	Ritonavir	Norvir	With darunavir and atazanavir – 100 mg QD with lopinavir – 200 mg QD		Cytochrome P450 (CYPP450) 3A4 Inhibitor
EI	Enfuvirtide	Fuzeon	Subcutaneous 90mg BID	Fatigue, insomnia, diarrhea, nausea	
EI	Maraviroc	Selzentry	300 mg BID		
INSTI	Cabotegravir	Vocabria	30 mg QD in combo with oral rilpivirine		
INSTI	Dolutegravir	Tivicay	50 mg	Psychiatric and neurological symptoms	
INSTI	Raltegravir	Isentress	400 mg BID	Diarrhea, nausea, headache, rare: hepatorenal damage	
EI	Fostemsavir	Rukobia	600 mg BID		Should not be taken with cytochrome P450 inducers - decreases the level
Post attachment inhibitor	Ibalizumab-Uiyk	Trogarzo	2 g as a single dose followed by a maintenance dose of 800 every 14 days after		
Capsid inhibitor	Lenacapavir	Sunlenca	2-day initiation, day 1: 600 mg once and 927 mg SubQ IM once, day 2: 600 mg once; 15-day initiation, day 1: 600 mg once, day 2: 600 mg once, day 8: 300 mg once, day 15: 927 mg SubQ IM once; maintenance dose: 927 mg SubQ IM every six months from the date of last injection +/- 2 weeks	Local injection site reaction	
PKE	Cobicistat	Tybost	150 mg QD with atazanavir or darunavir	Hepatotoxic – hyperbilirubinemia	CYP450 3A4 Inhibitor

**Table 2:** Combination-tablet FDA-approved HIV medications.

Medication name	Brand
Abacavir and lamivudine	Epzicom
Abacavir, dolutegravir, and lamivudine	Triumeq
Abacavir, zidovudine, and lamivudine	Trizivir
Atazanavir and cobicistat	Evotaz
Bictegravir, emtricitabine, and tenofovir alafenamide	Biktarvy
Cabitegravir and rilpivirine	Cabenuva
Darunavir and cobicistat	Prezcobix
Darunavir, cobicistat, emtricitabine, and tenofovir alafenamide	Symtuza
Dolutegravir and lamivudine	Dovato
Dolutegravir and rilpivirine	Juluca
Doravirine, lamivudine, and tenofovir disoproxil fumarate	Delstrigo
Efavirenz, emtricitabine, and tenofovir disoproxil fumarate	Atripla
Efavirenz, lamivudine, and tenofovir disoproxil fumarate	Symfi
Elvitegravir, cobicistat, emtricitabine, and tenofovir alafenamide	Genvoya
Elvitegravir Cobicistat, emtricitabine and tenofovir disoproxil fumarate	Stribild
Elvitegravir, rilpivirine, and tenofovir alafenamide	Odefsey
Emtricitabine and tenofovir alafenamide	Descovy
Emtricitabine and tenofovir disoproxil fumarate	Truvada

Current HIV testing available

Currently, there are a variety of different tests that can be utilized for HIV testing, including enzyme-linked immunosorbent assay (ELISA). This test is used to detect the presence or absence of HIV antibodies via blood or saliva analysis for antibodies against the virus [15]. The drawback of ELISA testing is that there is a required window period, which means that a patient will need weeks to months following infection to produce antibodies that yield a positive resulting test. Even though ELISA testing has high sensitivity, it is not a sufficient test for detecting early HIV infection. In addition, combined antigen/antibodies immunoassays can be used to detect both the presence of antibodies against the virus and viral antigens; these are known as fourth-generation HIV tests. These tests are used to screen for the p24 antigen, IgG/IgM anti-HIV-1/HIV-2 antibodies, as well as HIV-1 major subtype groups O, M, and N [16]. Nucleic acid-based tests (NATs) are also used in HIV testing to detect HIV-1 and HIV-2 infections. These tests utilize DNA polymerase chain reaction (PCR) tests to detect integrated viral DNA and RNA reverse transcription PCR (RT-PCR) to detect viral RNA [17]. These tests allow for the more rapid diagnosis of HIV as they target the actual viral code as opposed to the ELISA discussed above, which relies on antibody production. There are also HIV-1/HIV-2 differentiation assays used to differentiate between the diagnosis of HIV-1 and HIV-2. The two FDA-approved differentiation immunoassays currently include the GEENius HIV1/2 differentiation assay (Bio-Rad, Hercules, California, United States) and the VioOne HIV Profile supplemental assay (Avioq, Inc., Research Triangle Park, North Carolina, United States). Current CDC recommendations for testing include the use of an initial screening antigen/antibody immunoassay followed by an HIV-1/HIV-2 differentiation immunoassay with NAT testing [18]. Finally, there are self-testing kits available that utilize saliva, dried blood, and finger prick tests approved by the CDC in an effort to increase testing among a population and also decrease the healthcare burden [19].

Current safety precautions

Current safety precautions in place at hospitals are designed to decrease the incidence of and risks associated with needle stick injuries. In this regard, the most important precaution in place is education on best practices to prevent a needle stick injury. Occupational Safety and Health Administration (OSHA) protocol indicates that complementary training must be offered to all employees before the start and annually after. Training includes information about bloodborne pathogens and presentation of disease, risk, modes of transfer of bloodborne pathogens, explanation of the exposure control plan for the employer, information about high-risk exposure tasks, and postexposure prophylaxis with medical evaluation. Included in this training and beyond is the explanation of the selection of personal protective equipment (PPE), the proper use of PPE, and the removal and handling of PPE. OSHA also requires that PPE, including gloves, masks, and gowns, be readily available for staff to use in situations where there is potential for contact with a patient’s bodily fluids, blood, or contaminated equipment. In addition to glove use, hand hygiene and cleaning is crucial for minimizing the infection. CDC guidelines for proper hand hygiene recommend the use of alcohol-based hand rub with at least 60% ethanol or 70% isopropyl alcohol or hand soap with water for at least 20 seconds before and following the use of PPE [20]. Proper handling of needles is also crucial to mitigate risks of needlestick injuries and the spread of blood-borne pathogens. Precautions are also in place through proper training in needle use. Used needles should not be recapped and used sharps should be discarded following use by the user. Sharp containers should serve as closeable, puncture-resistant, leakproof containers present in every patient room to serve as a reservoir to dispose of needles and prevent unnecessary presence and risk of puncture. Further advancements have been made in safety precautions with safety-engineered syringes. These include syringes made with shields or syringes with retractable needs that retract following use [21].

Initial HIV infection

 Primary prevention, or preventing contact with infected blood and body fluids, is the most important strategy to avoid occupationally acquired HIV infection [22]. If occupational exposure occurs, appropriate postexposure protocols are crucial to maximize workplace safety and minimize infection [23]. Postexposure prophylaxis (PEP) for occupational exposures to HIV is comprised of antiretroviral therapy. Since 1990, the CDC has encouraged the use of optimal antiretroviral therapy as an HIV PEP regimen [24]. Medications included in an HIV PEP regimen should be selected to optimize side effects and toxicity profiles and a convenient dosing schedule [25]. Rapid determination of source patient HIV status provides essential information about the need to initiate or continue PEP. Many tests can be used to assess HIV status, such as rapid assays, third-generation chemiluminescent immunoassays, and fourth-generation combination p24 antigen-HIV antibody tests [25-30]. Administration of PEP should not be delayed while waiting for test results. If the source patient is determined to be HIV-negative, PEP should be discontinued, and no follow-up HIV testing for the occupationally exposed is indicated [25].

PEP should be initiated immediately for occupational exposures to HIV and should be administered for four weeks if source patient tests return HIV-positive and medications are tolerated with repeat testing occurring at six weeks, three months, and six months postexposure [31]. For all occupational exposures to HIV, a regimen consisting of three antiretroviral drugs, or more, is routinely recommended. An example regimen could contain two NRTI medication backbones and an integrase strand transfer inhibitor, a protease inhibitor, or an NNRTI [32]. In the circumstances of drug-resistant HIV strands or reduced regimen tolerance, other antiretroviral combinations can be utilized after consultation with an infectious disease expert [33]. The preferred HIV PEP regimen is Truvada 1 tablet orally once daily (tenofovir disoproxil fumarate (Viread) 300 mg + emtricitabine (FTC, Emtriva) 300 mg) plus raltegravir (RAL, Isentress) 400 mg orally twice daily. The following antiretroviral agents should be used for PEP only with expert consultation: abacavir (ABC, Ziagen), efavirenz (EFV, Sustiva), enfuvirtide (T20, Fuzeon), fosamprenavir (FOSAPV, Lexiva), maraviroc (MVC, Selzentry), saquinavir (SQV, Invirase), and stavudine (d4T, Zerit). The following agents are generally not recommended for PEP: didanosine (ddI, Videx EC), nelfinavir (NFV, Viracept), and tipranavir (d4T, Aptivus). Nevirapine is contraindicated for PEP [25,34]. If modifications to therapy are deemed appropriate due to adverse side effects or drug resistance, the PEP regimen can be altered after initiation. Re-evaluation of occupationally exposed persons should occur within 72 hours postexposure [25]. Postexposure follow-up appointments and testing are important to ensure the efficiency of the treatment regimen, to evaluate HIV viral load, and to prevent infection [35]. 

Who is at risk for HIV? 

Sexually active individuals and IV drug users are known to be at a high risk of HIV infection [36]. HIV, and other blood-borne pathogens, are a risk for those handling needles in healthcare settings. Occupational exposures to blood-borne pathogens remain a serious public health concern, with an estimated 385,000 needlestick and other sharps-related injuries in hospital-based healthcare personnel (HCP), with similar injuries across other healthcare settings [23]. With the prevalence of HIV in the general population estimated to be about 0.4%, the risk of contact with the virus in a healthcare setting is relatively high, Following percutaneous exposure to HIV in healthcare personnel, the risk of transmission is about 23% (95% CI, 0.0%-0.46%) [37,38]. In addition to percutaneous transmission, the blood-borne virus HIV can be transmitted via blood or contaminated fluid exposure of mucous membranes, nonintact skin, or human bites [23]. In the United States, 58 confirmed and 150 possible cases of occupationally acquired HIV infection were reported to the CDC between 1985 and 2013 [39]. Those with possible exposure are immediately started on antiretroviral therapy and reviewed for a baseline status of health in case infection is transmitted. This baseline physical exam and history can be used to assess the exposed patient’s health during treatment, alongside viral load assays [40].

Multiple factors may affect the risk of HIV transmission after occupational exposure. In a retrospective case-control study with healthcare personnel who had percutaneous exposure to HIV, exposure to a larger quantity of blood from the source patient and source patients with a terminal illness led to an increased risk for HIV infection. The larger quantity of infected blood could result from contact with a device visibly contaminated with source blood, a procedure that involves the direct placement of a needle into an artery or vein, or a deep injury. Together, these two factors suggest that the larger the viral load from the source patient, the higher the risk for infection [31].

Many comorbidities in patients who have been infected with HIV have been identified to increase the burden of HIV and decrease the quality of life. Alcohol use should be discouraged in patients with HIV as it is known to increase the risk of hepatotoxicity and fibrosis progression [41]. Patients living with HIV with lung disease (with HIV and co-existing lung disease), specifically chronic obstructive pulmonary disease and lung cancer, have an increased burden of disease (morbidity) and a poorer survival rate [42]. Living with a diagnosis of HIV while experiencing certain demographic and clinical characteristics, such as binge drinking, compulsive sexual behavior, polysubstance use, intimate partner violence, and depression, have a significantly decreased quality of life [43]. Lastly, HIV patients with vasculitides developed more vascular complications, responded less to antiretroviral therapy, and had worse outcomes when compared with non-HIV patients with vasculitides [44].

It has been found that the average risk of HIV infection after a needlestick injury leading to exposure to the blood of an HIV-infected individual is 0.3%. This means about one in every 1000 who are accidentally stuck will develop an infection [45]. However, it is important to note that injury with needlestick may not occur immediately following the needle use. HIV has been known to survive up to 42 days in syringes. Therefore, if they are improperly disposed of, they run the risk of infecting individuals even after the initial encounter of the needlestick directly with the HIV-infected patient. The risk of obtaining HIV after an accidental needle stick injury increases with the amount of blood as well as the concentration of the virus in that blood. It is also important to consider how deep the needle penetrates the skin of the individual, with deeper penetration leading to a higher chance of HIV infection [46]. 

MDCalc (New York, New York, United States), a resource used by many medical students, residents, and physicians has put out a useful tool called HIV Needle Stick Risk Assessment Stratification Protocol (RASP) that quantifies HIV exposure risk and the need for prophylaxis. This risk calculator assesses four main categories including: source population, inoculum type, method of transmission, and volume of inoculum. Prophylaxis is always recommended in the following situation: Source population contains known HIV+ patients with acute AIDS illness, fresh blood inoculum type, and IV transmission, with a massive volume of inoculum (an example is transfusion). In contrast, the lowest recommendation for prophylaxis with a percentage of less than 0.001% is a patient with unknown HIV status in a low-risk situation, inoculum with low-risk secretions such as tears, urine, or salvia, intact skin transmission, with a trace surface of volume (suture needle) [47].

Discussion

Accidental needle stick injuries are very common, estimated to amount to nearly 600,000 annually in the United States, with half not reported, and for the most part, they are avoidable. In the United States, the majority of people who have developed HIV as a result of needlestick injuries have worked in the medical field, including nurses, laboratory workers, or non-surgical physicians. An HIV infection is systemic and first suppresses the immune system but chronically can involve every organ in the body [1]. HIV is a retroviridae family-positive sense RNA virus that can synthesize DNA via reference transcriptase to infect the host cell genome [2]. HIV establishes an infection by infecting a CD4 T-cell on mucosal surfaces [3]. HIV can be spread by individuals after coming into contact with blood, urine, semen, and rectal or vaginal secretions. Individuals are commonly infected with HIV through vertical or horizontal transmission, where vertical transmission is from mother to fetus, and horizontal is from person to person contact through bodily fluids or contact with objects infected with HIV.

Although medication is available now for HIV patients, HIV will never be cured. For the treatment of HIV, over 30 antiretroviral drugs are classified based on their mechanism of action. The classifications currently include NRTIs, NNRTIs, PIs, NSTIs, an FI, and two EIs, one blocking chemokine receptor CCR5, and the other inhibiting viral interaction with CD4 [10]. The current cocktail treatment for HIV includes drugs with many different mechanisms of action. HIV treatment medications have side effects of nausea, vomiting, and diarrhea, while others have more extreme side effects, like hyperpigmentation, neurological symptoms, or liver injury. Various tests are used for HIV testing, including ELISA, combined antigen/antibodies immunoassay, nucleic acid-based tests, and HIV1/HIV2 differentiation assay [15-17]. Current CDC recommendations for testing include the use of an initial screening antigen/antibody immunoassay followed by an HIV1/HIV2 differentiation immunoassay with nucleic acid-based tests. Also, a self-test kit is available for the population to test themselves if they are at risk [19].

While patients with HIV should not be stigmatized by healthcare workers due to the low number of reported HIV-associated needle stick injuries, these healthcare workers need to take primary precautions in preventing transmission of HIV by using proper PPE when working with individuals with HIV as well as practicing sharps safety. WIth proper education, training, and steps in place if an accidental needle stick injury were to occur, the hope is to minimize accidental needle stick injuries and HIV exposure. It is important for all healthcare workers to accurately report needle stick injuries to provide accurate information to prospective needlestick injury HIV transmission. All HIV testing guidelines should also be followed in order to ensure that there are no new cases of healthcare workers infected with HIV after an accidental needle stick injury and that they receive proper treatment. 

## Conclusions

Many cases of HIV infections through needle sticks are accidental and avoidable. Hospital safety precautions are designed to decrease the incidence and risk associated with needle stick injuries. In order to minimize these accidental needle stick injuries, hospitals have begun to implement training, education, and protocols. Hospitals are also beginning to require that PPE including gloves, masks, and gowns be available for individuals for potential contact with infected patients. A large focus on current safety precautions is dependent on safe needle use and minimization of error.

The most important way to prevent HIV infection is to first use safety precautions. Postexposure prophylaxis for occupational exposures to HIV consists of antiretroviral therapy. For treatment, postexposure prophylaxis should be started immediately and continued for four weeks if the patient’s test is HIV positive. Sexually active individuals and IV drug users are at a higher risk of getting HIV infections and other blood-borne pathogens. Another high-risk group is individuals who have occupational exposure to needles or other sharps. Transmission through healthcare personnel also can increase the risks of individuals getting HIV.

## References

[1] King KC, Strony R (2024). Needlestick. StatPearls [Internet].

[2] Sued O, Grosso TM (2023). Pathophysiology of HIV and strategies to eliminate AIDS as a public health threat. Viral Infections and Antiviral Therapies.

[3] Zayas JP, Mamede JI (2022). HIV Infection and Spread between Th(17) Cells. Viruses.

[4] Aiken C, Rousso I (2021). The HIV-1 capsid and reverse transcription. Retrovirology.

[5] Wyżgowski P, Rosiek A, Grzela T, Leksowski K (2016). Occupational HIV risk for health care workers: risk factor and the risk of infection in the course of professional activities. Ther Clin Risk Manag.

[6] Nasirian M, Kianersi S, Karamouzian M, Sidahmed M, Baneshi MR, Haghdoost AA, Sharifi H (2020). HIV modes of transmission in Sudan in 2014. Int J Health Policy Manag.

[7] Pereira MC, Mello FW, Ribeiro DM (2023). Occupational HIV Transmission and
Prevention among Health Care Workers. J Dent.

[8] Patel P, Borkowf CB, Brooks JT, Lasry A, Lansky A, Mermin J (2014). Estimating per-act HIV transmission risk: a systematic review. AIDS.

[9] Bloodborne Pathogens and Needlestick Prevention. https://www.osha.gov/bloodborne-pathogens/evaluating-controlling-exposure.

[10] Yuan NY, Kaul M (2021). Beneficial and adverse effects of cART affect neurocognitive function in HIV-1 infection: balancing viral suppression against neuronal stress and injury. J Neuroimmune Pharmacol.

[11] Whiteley LB, Olsen EM, Haubrick KK, Odoom E, Tarantino N, Brown LK (2021). Correction to: a review of interventions to enhance HIV medication adherence. Curr HIV/AIDS Rep.

[12] (2023). FDA-Approved HIV Medicines. https://hivinfo.nih.gov/understanding-hiv/fact-sheets/fda-approved-hiv-medicines.

[13] Scarsi KK, Havens JP, Podany AT, Avedissian SN, Fletcher CV (2020). HIV-1 integrase inhibitors: a comparative review of efficacy and safety. Drugs.

[14] (2022). Chinese guidelines for the diagnosis and treatment of HIV/AIDS (2021 Edition). Infect Dis Immun.

[15] Farzin L, Shamsipur M, Samandari L, Sheibani S (2020). HIV biosensors for early diagnosis of infection: the intertwine of nanotechnology with sensing strategies. Talanta.

[16] Krasowski MD, Wier D, Smith S, Riedel A, Lauseker-Hao Y, Kelner M, Wang S (2021). Real-world clinical performance evaluation of a fourth-generation HIV antigen/antibody differentiation test. J Appl Lab Med.

[17] Ochodo EA, Guleid F, Deeks JJ, Mallett S (2021). Point-of-care tests detecting HIV nucleic acids for diagnosis of HIV-1 or HIV-2 infection in infants and children aged 18 months or less. Cochrane Database Syst Rev.

[18] Duncan D, Duncan J, Kramer B (2021). An HIV diagnostic testing algorithm using the cobas HIV-1/HIV-2 qualitative assay for HIV type differentiation and confirmation. J Clin Microbiol.

[19] O'Byrne P (2021). HIV self-testing: a review and analysis to guide HIV prevention policy. Public Health Nurs.

[20] Denault D, Gardner H (2024). OSHA bloodborne pathogen standards. StatPearls [Internet].

[21] Huang HM, Chien HC, Lin WL, Chang CH, Chang MY, Su JY, Mu PF (2022). Prevention of needle-stick injury among nurses in an acute ward of a hospital: a best practice implementation project. JBI Evid Implement.

[22] Siegel JD, Rhinehart E, Jackson M, Chiarello L (2007). 2007 guideline for isolation precautions: Preventing transmission of infectious agents in health care settings. Am J Infect Control.

[23] Shenoy ES, Weber DJ (2021). Occupational health update: evaluation and management of exposures and postexposure prophylaxis. Infect Dis Clin North Am.

[24] (1990). Public Health Service statement on management of occupational exposure to human immunodeficiency virus, including considerations regarding zidovudine postexposure use. MMWR Recomm Rep.

[25] Kuhar DT, Henderson DK, Struble KA (2013). Updated US Public Health Service guidelines for the management of occupational exposures to human immunodeficiency virus and recommendations for postexposure prophylaxis. Infect Control Hosp Epidemiol.

[26] Martin EG, Cornett J, Mohammed DY, Salaru G (2020). Rapid testing algorithm performance in a low-prevalence environment. Sex Transm Dis.

[27] Masciotra S, McDougal JS, Feldman J, Sprinkle P, Wesolowski L, Owen SM (2011). Evaluation of an alternative HIV diagnostic algorithm using specimens from seroconversion panels and persons with established HIV infections. J Clin Virol.

[28] Chavez P, Wesolowski L, Patel P, Delaney K, Owen SM (2011). Evaluation of the performance of the Abbott ARCHITECT HIV Ag/Ab Combo Assay. J Clin Virol.

[29] Lagatie O, Lauwers D, Singh H (2023). Towards novel HIV-1 serodiagnostic tests without vaccine-induced seroreactivity. Microbiol Spectr.

[30] Pitasi MA, Patel SN, Wesolowski LG, Masciotra S, Luo W, Owen SM, Delaney KP (2020). Performance of an alternative laboratory-based HIV diagnostic testing algorithm using HIV-1 RNA viral load. Sex Transm Dis.

[31] Cardo DM, Culver DH, Ciesielski CA (1997). A case-control study of HIV seroconversion in health care workers after percutaneous exposure. Centers for Disease Control and Prevention Needlestick Surveillance Group. N Engl J Med.

[32] Llibre JM, Brites C, Cheng CY (2023). Efficacy and safety of switching to the 2-Drug regimen Dolutegravir/Lamivudine versus continuing a 3- or 4-Drug regimen for maintaining virologic suppression in adults living with human immunodeficiency virus 1 (HIV-1): week 48 results from the phase 3, noninferiority SALSA randomized trial. Clin Infect Dis.

[33] Ávila-Ríos S, Parkin N, Swanstrom R, Paredes R, Shafer R, Ji H, Kantor R (2020). Next-generation sequencing for HIV drug resistance testing: Laboratory, clinical, and implementation considerations. Viruses.

[34] Phanuphak N, Gulick RM (2020). HIV treatment and prevention 2019: current standards of care. Curr Opin HIV AIDS.

[35] Huynh K, Kahwaji CI (2024). HIV testing. StatPearls [Internet].

[36] Barbanotti D, Tincati C, Tavelli A (2023). HIV-indicator condition guided testing in a hospital setting. Life (Basel).

[37] Chapman LE, Sullivent EE, Grohskopf LA (2008). Postexposure interventions to prevent infection with HBV, HCV, or HIV, and tetanus in people wounded during bombings and other mass casualty events--United States, 2008: recommendations of the Centers for Disease Control and Prevention and Disaster Medicine and Public Health Preparedness. Disaster Med Public Health Prep.

[38] Baggaley RF, Boily MC, White RG, Alary M (2006). Risk of HIV-1 transmission for parenteral exposure and blood transfusion: a systematic review and meta-analysis. AIDS.

[39] Joyce MP, Kuhar D, Brooks JT (2015). Notes from the field: occupationally acquired HIV infection among health care workers - United States, 1985-2013. MMWR Morb Mortal Wkly Rep.

[40] Saag MS (2021). HIV infection - screening, diagnosis, and treatment. N Engl J Med.

[41] Osna NA, Ganesan M, Seth D, Wyatt TA, Kidambi S, Kharbanda KK (2021). Second hits exacerbate alcohol-related organ damage: an update. Alcohol Alcohol.

[42] Leung JM (2023). HIV and chronic lung disease. Curr Opin HIV AIDS.

[43] de Oliveira Gomes M, Castro R, Corrêa da Mota J, De Boni RB (2023). Association of syndemic conditions and quality of life among people living with HIV/AIDS. AIDS Care.

[44] Vega LE, Espinoza LR (2020). Vasculitides in HIV Infection. Curr Rheumatol Rep.

[45] (2023). Exposure to Blood. https://www.cdc.gov/hai/pdfs/bbp/exp_to_blood.pdf.

[46] (2008). Needle stick injuries in the community. Paediatr Child Health.

[47] Les Vertesi (2023). HIV Needle Stick Risk Assessment Stratification Protocol (RASP). MDCALC.

